# UV-induced proteolysis of RNA polymerase II is mediated by VCP/p97 segregase and timely orchestration by Cockayne syndrome B protein

**DOI:** 10.18632/oncotarget.14205

**Published:** 2016-12-26

**Authors:** Jinshan He, Qianzheng Zhu, Gulzar Wani, Altaf A. Wani

**Affiliations:** ^1^ Department of Radiology, The Ohio State University, Columbus, OH 43210, USA; ^2^ Department of Molecular and Cellular Biochemistry, The Ohio State University, Columbus, OH 43210, USA; ^3^ James Cancer Hospital and Solove Research Institute, The Ohio State University, Columbus, OH 43210, USA

**Keywords:** RNA polymerase II, valosin-containing protein, ubiquitin, cockayne syndrome B, von hippel-lindau tumor suppressor protein

## Abstract

RNA polymerase II (RNAPII) acts as a damage sensor for transcription-coupled nucleotide excision repair (TC-NER) and undergoes proteolytic clearance from damaged chromatin by the ubiquitin-proteasome system (UPS). Here, we report that Valosin-containing protein (VCP)/p97, a druggable oncotarget, is essential for RNAPII's proteolytic clearance in mammalian cells. We show that inhibition of VCP/p97, or siRNA-mediated ablation of VCP/p97 and its cofactors UFD1 and UBXD7 severely impairs ultraviolet radiation (UVR)-induced RNAPII degradation. VCP/p97 interacts with RNAPII, and the interaction is enhanced by Cockayne syndrome B protein (CSB). However, the VCP/p97-mediated RNAPII proteolysis occurs independent of CSB. Surprisingly, CSB enhances UVR-induced RNAPII ubiquitination but delays its turnover. Additionally, VCP/p97-mediated RNAPII turnover occurs with and without Von Hippel-Lindau tumor suppressor protein (pVHL), a known substrate receptor of Elongin E3 ubiquitin ligase for RNAPII. Moreover, pVHL re-expression improves cell viability following UVR. Whereas, VCP/p97 inhibition decreases cell viability and enhances a low-dose UVR killing in presence of pVHL. These findings reveal a function of VCP/p97 segregase in UVR-induced RNAPII degradation in mammalian cells, and suggest a role of CSB in coordinating VCP/p97-mediated extraction of ubiquitinated RNAPII and CSB itself from chromatin.

## INTRODUCTION

Transcription is one of fundamental processes in living cells. Transcriptional elongation by RNA polymerase II (RNAPII) often encounters obstacles, including DNA damage, chromatin structures and molecular machineries of other DNA-templated processes, which can cause elongating RNAPII to stall or arrest. The irreversibly arrested RNAPII, e.g., at ultraviolet radiation (UVR)-induced photolesions, can become an obstruction to DNA replication and DNA repair machineries and is highly deleterious to cells. In yeast, so far, proteolysis of RNAPII by the ubiquitin-proteasome system (UPS) has been shown to resolve stalled RNAPII [1].

Proteolysis of RNAPII is a tightly regulated multistep process involving ubiquitination of RNAPII, release of the ubiquitin conjugates from chromatin, and proteolytic processing by proteasome. In yeast, ubiquitination of Rpb1, the large subunit of RNAPII, is conducted by a cooperative action of Rsp5 E3 ubiquitin ligase [2] and an Elongin ubiquitin ligase complex, which contains Ela1 (Elongin A), Cul3, Elc1 (Elongin C) and Rbx1 [3, 4]. In mammals, several E3 ubiquitin ligases including BRCA1-BARD ligase [5, 6], Nedd4 ligase [7] and mammalian counterpart Elongin ubiquitin ligase complex [8–11] were reported to ubiquitinate damage-arrested RNAPII. The Elongin ubiquitin ligase complex uses Von Hippel-Lindau (VHL) tumor suppressor protein (pVHL) as a substrate receptor for RNAPII ubiquitination [9]. It has been known that the pVHL protein directly binds Elongin BC complex and inhibits transcription elongation [12].

Release of ubiquitinated Rpb1 from chromatin in yeast requires the function of Cdc48-Ufd11-Npl4 segregase protein complex, its adaptor proteins Ubx4 and Ubx5 [13], as well as chromatin remodeler INO80 [14]. CDC48 protein, also known as Valosin-containing protein (VCP)/p97, is a member of an evolutionally conserved AAA (ATPase-associated with various cellular activities) family [15]. CDC48 plays a well-established role in segregating ubiquitinated substrates from protein complexes, aggregates, membranes and chromatin [16–18]. VCP/p97 cooperates with different sets of mutually exclusive cofactors and adaptors for different cellular functions [19]. The cofactor UFD1 and NPL4 are the known VCP/p97 substrate-recruiting cofactors, which are needed for extracting the misfolded proteins from endoplasmic reticulum (ER) membrane during ER-associated degradation [20–23]. Yeast Ubx5 is a member of UBA-UBX protein family. Its mammalian counterpart protein UBXD7 is involved in VCP/p97-mediated extraction of ubiquitinated DNA damage sensors DDB2, XPC and Cockayne syndrome B protein (CSB) from damaged chromatin [24–26].

Lesion-stalled elongating RNAPII also triggers transcription-coupled repair processes. For example, photolesion-stalled RNAPII, together with CSB, initiates transcription-coupled nucleotide excision repair (TC-NER) [27, 28]. In the process of TC-NER, DNA lesion stabilizes the interaction between RNAPII with CSB [29, 30]. The RNAPII-CSB complex recruits Cockayne syndrome A protein (CSA), transcription factor II H (TFIIH) and other core NER factors, as well as non-NER factors to the lesion sites [31]. Once a lesion is verified by TFIIH, the stalled RNAPII along with other components of elongating machinery backtracks or is resolved, and the transcription elongation process is funneled into assembling preincision complex of NER.

How the stalled RNAPII, along with elongating machinery, at DNA lesion, is resolved in mammalian cells has not been assessed experimentally. The issue is complicated not only by the participation of multiple E3 ubiquitin ligases for RNAPII ubiquitination but also by the involvement of CSB and CSA in RNAPII ubiquitination and degradation [32, 33]. In this study, we have experimentally defined the role of VCP/p97 in UVR-induced RNAPII degradation in mammalian cells. We showed that VCP/p97-mediated UVR-induced RNAPII occurs regardless of the CSB status. We also examined the physical interaction between VCP/p97 and RNAPII, the UVR-induced RNAPII ubiquitination and turnover, as well as the role of CSB in these processes. We further provide evidence that UVR-induced degradation of RNAPII *via* the cooperative action of pVHL-containing Elongin E3 ubiquitin ligase and VCP/p97 is critical for cell viability following UVR.

## RESULTS

### VCP/p97 inhibition prevents UVR-induced degradation of RNAPII

**Figure 1:**
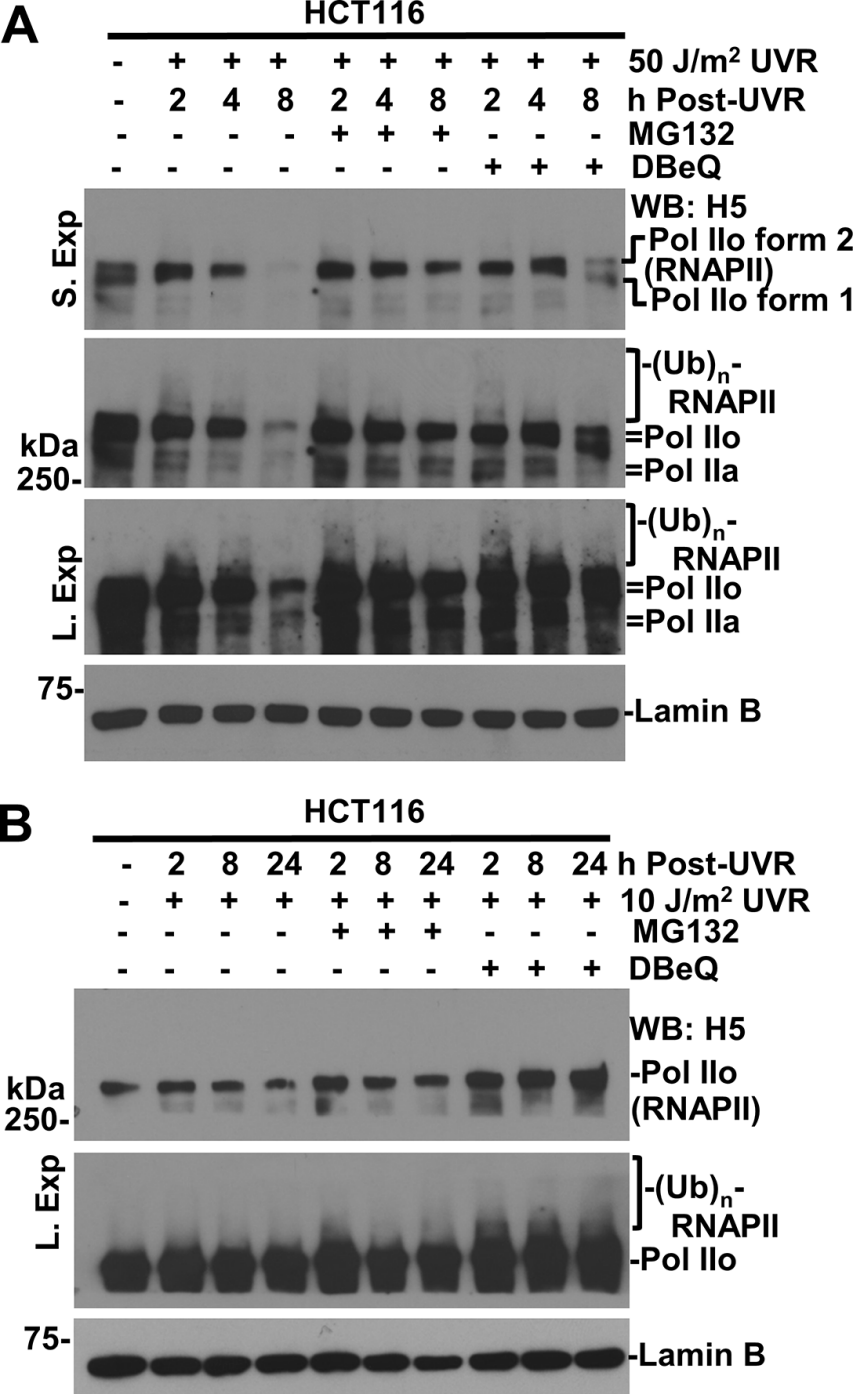
Inhibition of VCP/p97 impairs UV-induced RNAPII degradation (**A**) HCT116 cells were UV-irradiated at 50 J/m^2^, maintained for the indicated times with or without treatment of 10 μM MG132 or 10 μM VCP/p97 inhibitor DBeQ, and the cellular proteins were analyzed by Western blotting. Different exposures were used to show various forms of RNAPII as detected by indicated RNAPII antibody. “S. Exp.” indicates a shorter exposure and “L. Exp.” indicates a longer exposure in protein detection. Lamin B blot serves as a loading control. (**B**) HCT116 cells were UV-irradiated 10 J/m^2^; inhibitor treatments were the same as in Figure 1A.

To determine whether VCP/p97 is involved in UVR-induced degradation of RNAPII in mammalian cells, we first examined the effect of VCP/p97 inhibition on proteolysis of RNAPII by DBeQ, a specific small molecule inhibitor of VCP/p97 ATPase [34]. The RNAPII levels in HCT116 cells at 50 J/m^2^ exhibited a clear and progressive decrease from 2 to 8 h following UVR. Individual treatments of cells with DBeQ or MG132 prevented RNAPII degradation (Figure 1A). It is noteworthy that anti-RNAPII H5 antibody primarily recognizes Pol IIo forms, but the Pol IIa forms can be observed by optimizing gel separations and exposure times during Western blotting. These forms of RNAPII were essentially characterized and annotated in previous studies [35] and were further confirmed in this study by independent set of RNAPII antibodies (Supplementary Figure S1). The slow-migrating modified RNAPII forms were seen at 2 h regardless of the inhibitor treatment and the overall results were consistent with previous observations of RNAPII ubiquitination induced by UVR [35]. At 10 J/m^2^, UVR-induced RNAPII degradation was discernible but not prominent in HCT116 cells (Figure 1B). However, DBeQ elevated the levels of RNAPII. Because ubiquitin specific protease 7 (USP7) was implicated in protecting RNAPII from proteasomal degradation [36, 37], we compared the dynamics of RNAPII levels following UVR in parental HCT116 and HCT116-derived USP7 knockout cells (Supplementary Figure S1). The UVR-induced RNAPII degradation exhibited a similar dose-dependent response in both HCT116 and USP7-deficient cells. At 50 J/m^2^, MG132 and DBeQ also prevented RNAPII degradation in USP7-deficient HCT116 cells. Taken together, these results indicate that VCP/p97 is involved in ubiquitin-mediated RNAPII degradation regardless of the status of USP7.

### VCP/p97 functions in RNAPII degradation in the presence of CSB

**Figure 2:**
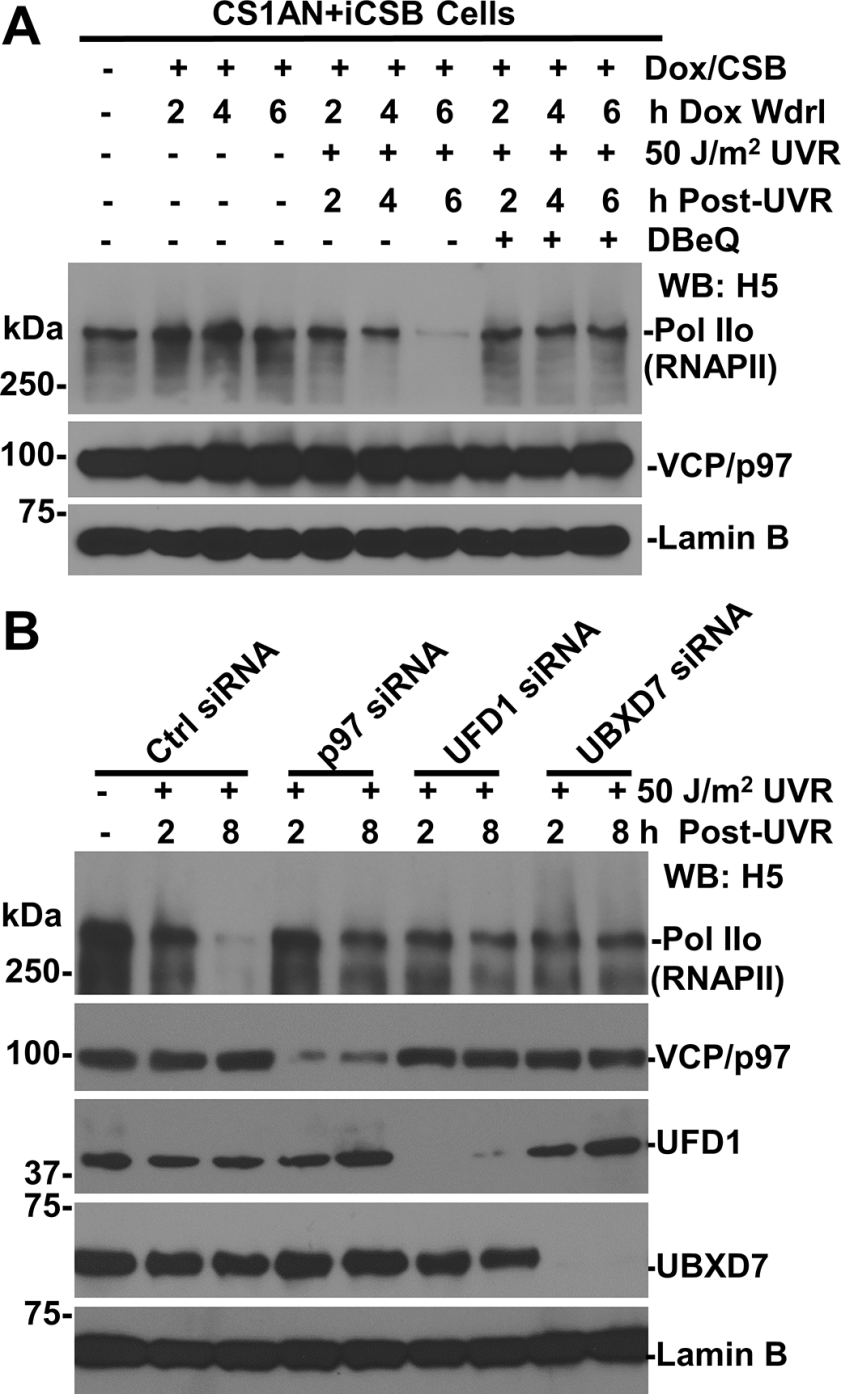
Function of VCP/p97, UFD1 and UBXD7 is required for UV-induced RNAPII degradation (**A**) Inhibition of VCP/p97 impairs UV-induced RNAPII degradation in corrected CSB-deficient cells. The CS1AN+iCSB cells were induced for CSB expression in presence of 1 μg/ml doxycycline (Dox) for 24 h. The cells were UV irradiated, treated with DBeQ and maintained under Dox withdrawal (Wdrl) for indicated post-UVR time periods. The RNAPII, CSB, and VCP/p97 in cell lysates were detected by Western blotting with specific antibodies. Lamin B blot serves as a loading control. (**B**) HCT116 cells were transfected with the control (Ctrl) or target specific siRNA, UV irradiated and maintained until the indicated time points. RNAPII, VCP/p97, UFD1 and UBXD7 in cell lysates were detected by Western blotting.

Because Cockayne syndrome proteins are suggested to play a role in UVR-induced ubiquitination and degradation of RNAPII [32, 33], we first investigated VCP/p97 function in RNAPII degradation in the presence of CSB by employing corrected CSB-deficient CS1AN cells, which harbor Doxycycline (Dox)-inducible CSB transgenes (Figure 2A). In these cells, the Dox-induced CSB expression and UV-induced CSB degradation were confirmed upon 24-h Dox induction and withdrawal (Figures 4A and 6A). More importantly, UV irradiation reduced the steady-state level of RNAPII in the presence of CSB. As expected, the loss of RNAPII was again prevented by VCP/p97 inhibition, indicating the loss of RNAPII requires VCP/p97 function.

**Figure 3:**
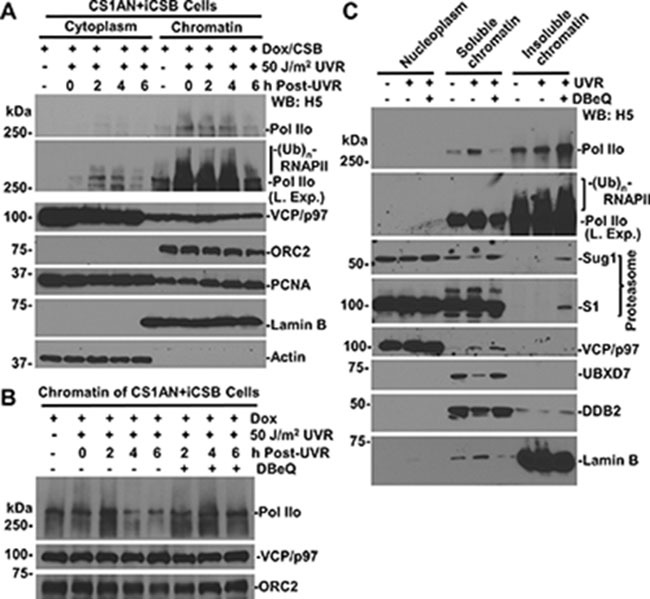
VCP/p97 inhibition prevents UV-induced RNAPII proteolysis from chromatin (**A**) CS1AN+iCSB cells were Dox-induced, UV irradiated, maintained for indicated time periods, and then subjected to cellular protein fractionation protocol. RNAPII, VCP/p97, Actin, Lamin B, and ORC2 and PCNA in fractions containing the same amount proteins were detected by Western blotting. Actin, Lamin B and nuclear protein ORC2 served as fractionation mark or as loading controls. “L. Exp.” indicates longer exposure. (**B**) The CS1AN+iCSB cells were induced for CSB expression and UV-irradiated as in Figure 3A, but were treated with DBeQ. The chromatin fractions were analyzed by Western blotting for RNAPII, ORC2 and VCP/p97. (**C**) HCT116 cells were pre-treated with 10 μM DBeQ or vehicle for 4 h, UV-irradiated at 50 J/m^2^ and kept under DBeQ treatment for additional 2 h. Nucleoplasmic, soluble chromatin and insoluble chromatin fractions were isolated by cellular protein fractionation. The RNAPII, 19S proteasomal Sug1 and S1, as well as UBXD7 were examined in indicated chromatin fractions containing the same amount of proteins. Lamin B served as a fractionation mark and a loading control.

**Figure 4:**
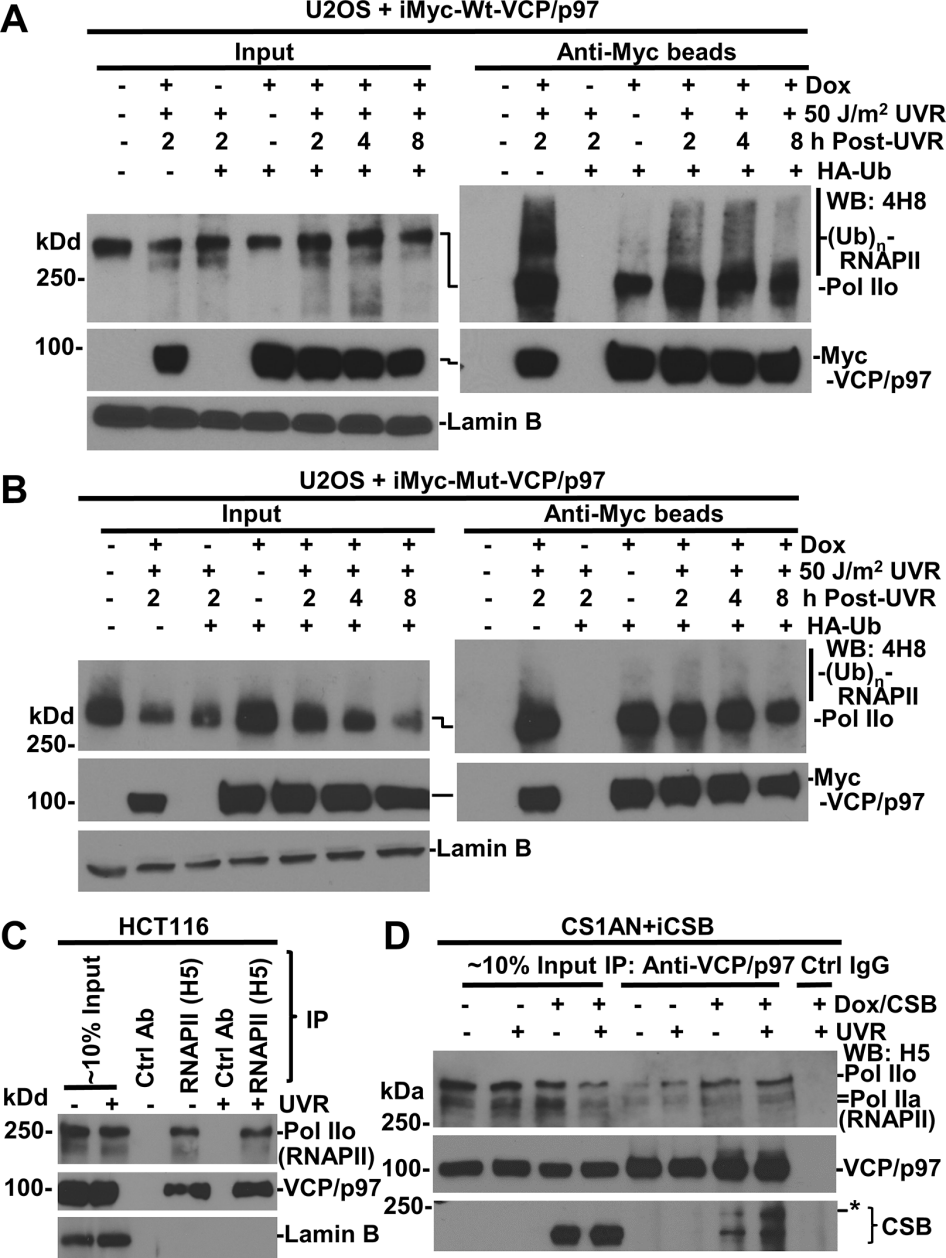
RNAPII interacts with VCP/p97 (**A**) U2OS cells, harboring inducible Myc-tagged-Wt-VCP/p97 transgenes (iMyc-Wt-VCP/p97), were transfected with expression constructs for HA-tagged ubiquitin (HA-Ub) for 48 h and in the second 24 h, Dox was added for transgene induction. The transfected cells were UV irradiated and maintained for indicated time. The cell lysates were made in RIPA buffer and subjected to immunoprecipitation. The RNAPII, VCP/p97 proteins in Input (~10%) and immunoprecipitates were detected by Western blotting. Lamin B blot served as the loading control. (**B**) U2OS cells harboring inducible Myc-tagged EQ-VCP/p97 transgenes (iMyc-Mut-VCP/p97) were used in the experiments similar to that in Figure 4A. (**C**) HCT116 cells were UV-irradiated at 20 J/m^2^, and the cell lysates in RIPA buffer were prepared 2 h later. The RNAPII, VCP/p97 and LaminB were detected in Input (~10%) and in immunoprecipitates recovered by RNAPII or control antibody. (**D**) CS1AN+iCSB cells were Dox-induced for CSB expression for 24 h, UV irradiated at 20 J/m^2^, maintained for 2 h, and then cell lysates were made in RIPA. The immunoprecipitation and Western blotting were carried out as in Figure 4C. *indicates a modified CSB species.

**Figure 5:**
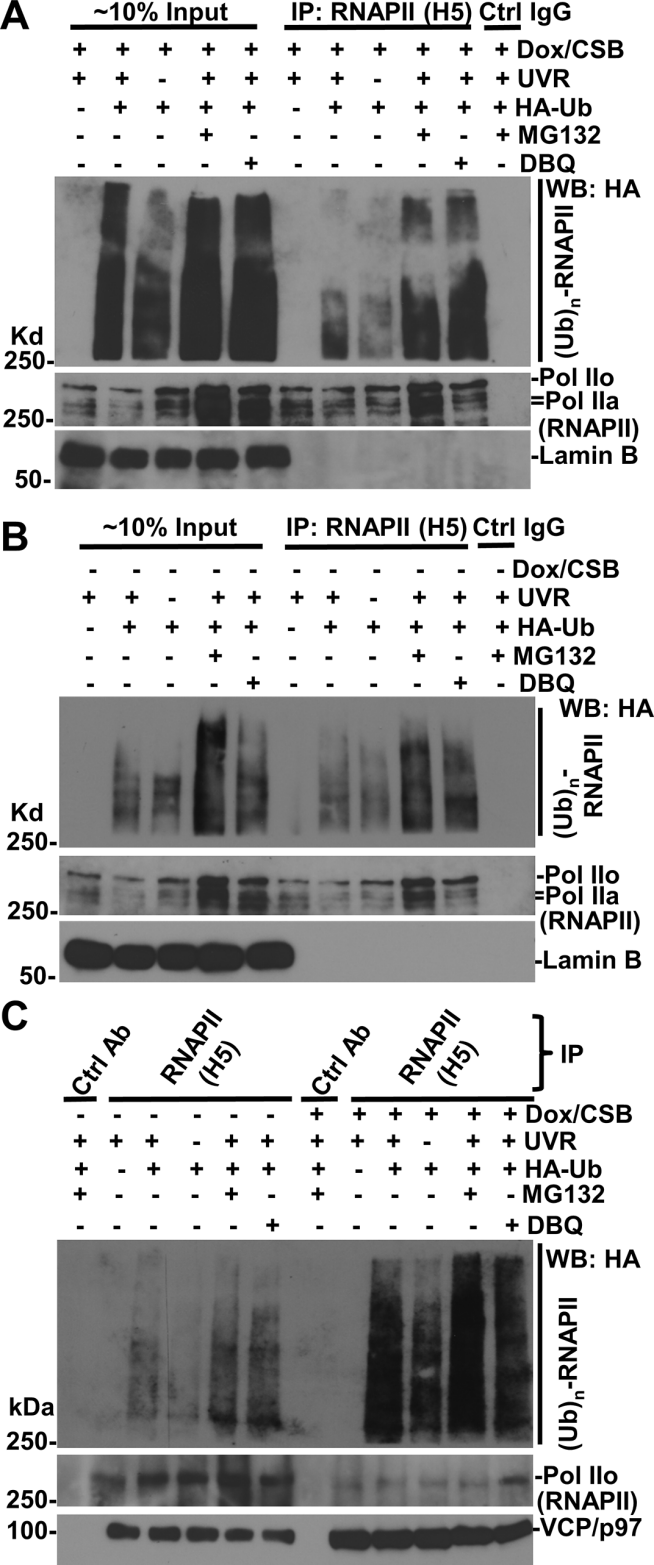
RNAPII ubiquitination occurs in the presence or the absence of CSB (**A**) CS1AN+iCSB cells were transfected with expression constructs for HA-tagged ubiquitin (HA-Ub) for 48 h, and Dox was added for CSB induction in the second 24 h. The transfected cells were UV irradiated at 50 J/m^2^, treated with MG132 or DBeQ for 4 h. The cell lysates were made in RIPA buffer and the immunoprecipitation was performed with RNAPII or control (Ctrl) antibodies. The Ub conjugates, RNAPII, Lamin B proteins in Input (~10%) and immunoprecipitates were detected by Western blotting. (**B**) CS1AN+iCSB cells were transfected UV-irradiated, and inhibitor-treated without CSB induction. The immunoprecipitation and Western blotting were performed the same as in Figure 5A. (**C**) Immunoprecipitation samples from experiments in Figure 5A and 5B were analyzed together in Western blotting for ubiquitin conjugates, RNAPII and VCP/p97.

**Figure 6:**
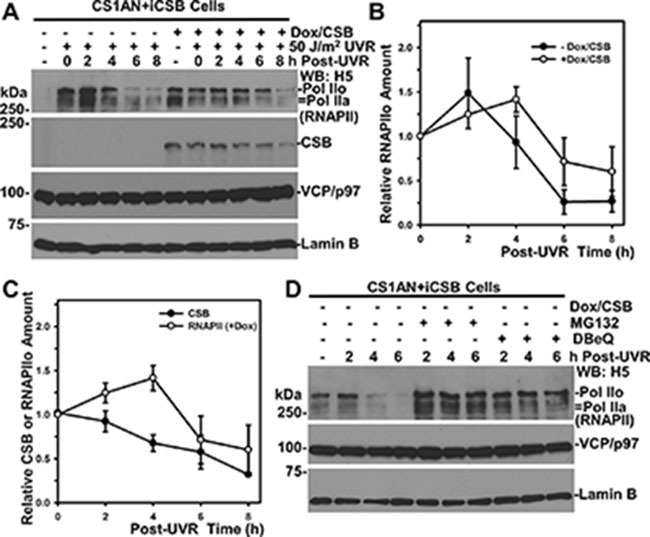
RNAPII degradation slows down in the presence of CSB (**A**) CS1AN+iCSB cells, with or without CSB induction, were UV irradiated at 50 J/m^2^ and maintained for indicated post-UV time periods. The RNAPII, CSB, VCP/p97 and Lamin B in cell lysates were detected by Western blotting. Lamin B blot served as loading control. (**B**) RNAPII blots from multiple (> 3) independent experiments as in Figure 6A were quantitatively examined by ImageJ software. The relative RNAPII amount was calculated on the basis of 0-h time point and plotted against time course. (**C**) CSB blots from multiple (> 3) independent experiments as in Figure 6A were quantitatively examined by ImageJ software. The relative CSB amount was calculated on the basis of 0-h time point and plotted together with RNAPII against time course. (**D**) VCP/p97 inhibition impairs RNAPII degradation in the absence of CSB. CS1AN+iCSB cells without CSB induction were UV irradiated at 50 J/m^2^, treated with MG132 or DBeQ and maintained for the indicated time period. The RNAPII, VCP/p97 and Lamin B in cell lysates were detected by Western blotting.

We next examined the effect of functional ablation of VCP/p97 segregase complex. The target specific siRNA but not the control siRNA depleted the corresponding VCP/p97, UFD1 and UBXD7 proteins (Figure 2B). As a result, the depletion of these components prevented UVR-induced RNAPII loss. Notably, UVR-induced RNAPII loss required VCP/p97 and the same UBXD7 adaptor for UVR-induced CSB degradation, which was also prevented by VCP/p97 inhibition [26]. Thus, the loss of CSB could not be the reason for the failure of RNAPII degradation. Given that the role of proteasome in RNAPII degradation, we concluded that VCP/p97 and UBXD7 adaptor are functionally required for ubiquitin-mediated RNAPII degradation.

### VCP/p97 segregase extracts RNAPII from chromatin

RNAPII is known to stall at DNA lesions scattered along chromatin. Therefore, we isolated cytoplasmic and chromatin fractions of CS1AN+iCSB cells to determine the existence of RNAPII in corresponding cellular protein fractions. As shown in Figure 3A, RNAPII was mainly present in chromatin fraction where it exhibited a gradual decrease following cellular UVR exposure. The slow-migrating modified RNAPII forms appeared in chromatin fraction upon UV irradiation. In comparison, only a small fraction of VCP/p97 was detectable in chromatin while the majority resided in cytoplasm. Correspondingly, proliferating cell nuclear antigen (PCNA) was detected in cytoplasm and to a lesser extent in chromatin. Whereas, DNA origin recognition complex 2 subunit (ORC2), a known chromatin-associated protein, resided exclusively in chromatin.

When the degradation of chromatin-associated RNAPII was examined, the results showed that as compared with DBeQ-untreated controls, the VCP/p97 inhibition apparently prevented the UVR-induced RNAPII loss from chromatin at 4 and 6 h post-UVR (Figure 3B).

We then determined the distribution of RNAPII in nucleoplasmic, soluble (nuclease-releasable) and insoluble chromatin fractions following 2 h of UVR under the conditions of VCP/p97 inhibition (Figure 3C). RNAPII was seen in soluble chromatin and to a higher extent in insoluble chromatin, indicating the tight association of RNAPII with chromatin. Although UV irradiation immobilized RNAPII to soluble chromatin fractions, the VCP/p97 inhibition and UV irradiation together further immobilized RNAPII into insoluble chromatin fraction, where the modified RNAPII forms were exclusively detected. The modified RNAPII forms were slightly elevated by UVR and DBeQ pre-treatments. The examination of proteasomal Sug1 and S1 proteins indicated that while VCP/p97 inhibition restored the UVR-depleted Sug1 in soluble chromatin, DBeQ immobilized both Sug1 and S1 in insoluble chromatin. Predictably, only a small fraction of VCP/p97 resides in soluble chromatin, and the presence was slightly enhanced by UVR and DBeQ treatment. Meanwhile, VCP/p97 inhibition restored the UVR-depleted UBXD7 adaptor in soluble chromatin. The accumulation of Sug1, S1 and UBXD7 in soluble or insoluble chromatin is presumably attracted by the unprocessed ubiquitin conjugates due to VCP/p97 inhibition. As a control, DDB2, a known VCP/p97 substrate [24], was seen to reside primarily in soluble chromatin, get depleted upon UVR exposure and, as expected, get restored by VCP/p97 inhibition. Collectively, these results indicated that VCP/p97 functions in extracting ubiquitinated lesion-stalled RNAPII, which is tightly associated with chromatin.

### RNAPII interacts with VCP/p97

We next carried out immunoprecipitation experiments to determine the interaction between RNAPII and VCP/p97. For this, we used genetically engineered U2OS cells harboring an inducible Myc-tagged wild-type (Wt) or mutant (Mut)-VCP/p97. The U2OS cells were transfected with expression construct for HA-tagged ubiquitin (HA-Ub) for 48 h with Dox added for the second 24-h period to enable Myc-tagged VCP/p97 expression. HA-ubiquitin expression allowed the examination of cellular ubiquitination. Our previous experiments have demonstrated the binding of VCP/p97 to ubiquitin conjugates, and ubiquitinated and native CSB protein [26]. As shown in Figure 4A, RNAPII was detected in anti-Myc immunoprecipitates from VCP/p97 induced cells. Moreover, the slow-migrating modified RNAPII showed a clear presence in anti-Myc immunoprecipitates from UV irradiated cells, indicating that VCP/p97 binds to both RNAPII and ubiquitinated RNAPII. The RNAPII-VCP/p97 interaction was further confirmed by similar experiments using another U2OS cell line, which harbors an inducible Myc-tagged EQ-VCP/p97 (Figure 4B). We persistently observed that ubiquitinated species in the EQ-VCP/p97-bound RNAPII were relatively less discernible. This could be explained by potential dominant negative effect of EQ-VCP/p97, which causes tight binding of ubiquitin-conjugated RNAPII species to chromatin and rendering it hard to extract in RIPA buffer. Nevertheless, the binding of VCP/p97 to RNAPII was authenticated by using RNAPII antibody for reciprocal immunoprecipitation (Figure 4C). Here, VCP/p97 was clearly detected by anti-RNAPII immunoprecipitation but not in corresponding controls.

We extended the immunoprecipitation analysis to CS1AN+iCSB cells with and without CSB induction (Figure 4D). Both RNAPII and CSB were present in the samples of immunoprecipitation with VCP/p97 antibody. Remarkably, CSB expression increased the abundance of RNAPII associated with VCP/p97. Of note, our previous results have shown the association of VCP/p97 with the UFD1 and NPL4 [26]. Thus, CSB promotes or enhances the association of VCP/p97 segregase complex with RNAPII.

### CSB enhances ubiquitination of RNAPII

We reasoned that the enhancement of the association of VCP/p97 with RNAPII by CSB might be resulted from RNAPII ubiquitination, which was previously shown to be somewhat dependent on functional CSA and CSB, the latter accelerates RNAPII ubiquitination during early post-UVR times [32]. Therefore, we further determined the effect of CSB on RNAPII ubiquitination. The CS1AN+iCSB cells were transfected with expression constructs for HA-Ub for 48 h and during the second 24-h period, Dox was added for CSB induction. In Figure 5A, ubiquitinated RNAPII species with low molecular masses showed a clear presence in UV-irradiated cells. More importantly, both MG132 and DBeQ significantly augmented the presence of ubiquitin-modified RNAPII species and increased the molecular mass higher, indicating that UVR-induced RNAPII ubiquitination and the accumulation of ubiquitin conjugates through the inhibitory actions on proteasome or VCP/p97.

In the absence of CSB, RNAPII-ubiquitin conjugates also exhibited an appearance (Figure 5B). A side-by-side comparison of RNAPII-ubiquitin conjugates, however, indicated that the RNAPII ubiquitination was much more apparent in the presence than that in the absence of CSB. This was true both with and without proteasome or VCP/p97 inhibition, arguing that these inhibitors only allow accumulation of RNAPII-ubiquitin conjugates but do not change the RNAPII ubiquitination status affected by CSB (Figure 5C). In addition, increased levels of VCP/p97 were detected in anti-RNAPII precipitates in the presence of CSB, further supporting the enhanced interaction of VCP/p97 with RNAPII by CSB. Taken together, these experiments indicated that RNAPII ubiquitination is enhanced by CSB, albeit CSB is not essential for RNAPII ubiquitination.

### RNAPII turnover is delayed by CSB

Although CSA and CSB proteins promote the rapid RNAPII ubiquitination upon UV irradiation [32], the rate of UVR-induced RNAPII turnover is not significantly affected by the functional status of CSA and CSB [38]. To understand this conundrum, we again utilized CS1AN+iCSB cells because the inducible CSB expression allows a comparison of UVR-induced RNAPII turnover within identical cellular backgrounds (Figure 6A). In the absence of CSB, the RNAPII was detectable at a lesser extent without UVR, due presumably to the altered RNAPII phosphorylation [38]. UVR-induced RNAPII degradation occurred regardless of CSB. However, comparison of the kinetics of UVR-induced RNAPII turnover clearly showed a delay of RNAPII turnover in the presence of CSB (Figure 6A and 6B). The UVR-induced RNAPII turnover occurred from 4 to 6 h period in the absence of CSB (Figure 6B). By contrast, in the presence of CSB, a clear RNAPII turnover is seen to begin from 6 to 8 h and with greater residual RNAPII. Moreover, UVR-induced CSB turnover steadily occurred following UVR up to 8 h (Figure 6C). The RNAPII and CSB turnover overlapped around the 4 to 8 h period. Taken together, we concluded that CSB slows down UVR-induced RNAPII turnover.

We further tested if UVR-induced RNAPII turnover without CSB requires the function of VCP/p97. Indeed, both MG132 and DBeQ prevented UVR-induced RNAPII turnover in the absence of CSB (Figure 6D). Thus, UVR-induced RNAPII turnover is mediated by VCP/p97 regardless of CSB, which increases RNAPII ubiquitination but delays RNAPII turnover.

### VCP/p97 inhibition impairs both pVHL-dependent and -independent RNAPII degradation and affects cell viability following UV irradiation

**Figure 7:**
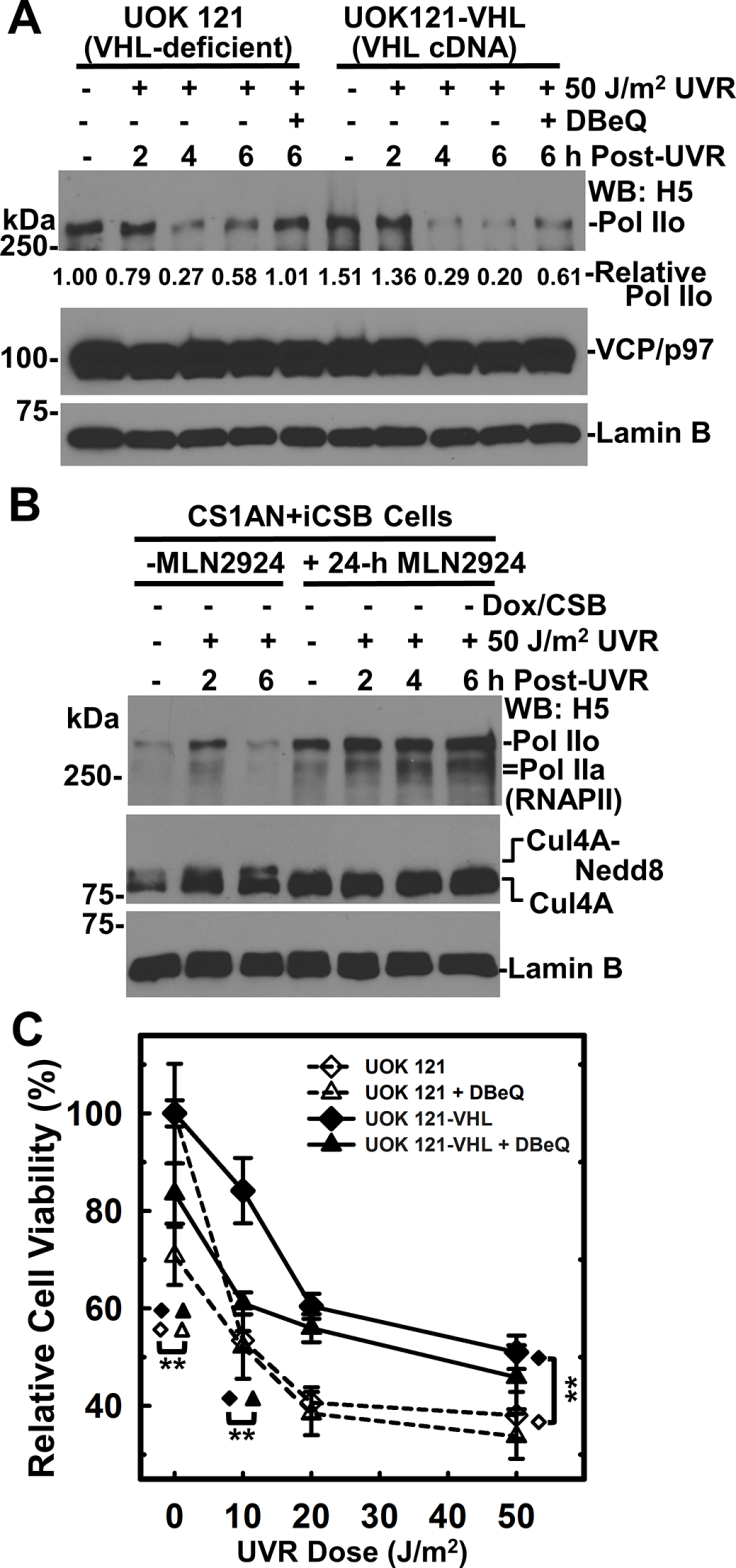
VCP/p97 inhibition impairs RNAPII degradation in the presence or absence of pVHL expression (**A**) pVHL-deficient UOK121 and the pVHL-proficient UOK121-VHL cells were UV irradiated at 50 J/m^2^, treated with 10 μM DBeQ and maintained for indicated time period. The RNAPII, VCP/p97 and Lamin B in cell lysates were detected by Western blotting. Lamin B blot served as loading control. Relative RNAPII amount is calculated based on quantitation with ImageJ software. (**B**) CS1AN+iCSB cells without CSB induction were pretreated with neddylation inhibitor MLN4924 at 1.2 μM for 24 h, UV irradiated at 50 J/m^2^ and maintained under neddylation inhibition for the indicated time period. The RNAPII and Cul4A in cell lysates were detected by Western blotting. Lamin B blot served as loading control. (**C**) UOK121 and UOK121-VHL cells were UV irradiated at doses from 10 to 50 J/m^2^, incubated with DBeQ at 5 μM or vehicle for 48 h, and cell viability was examined by MTT assay. The relative cell viability was calculated by normalizing against controls of each cell line without UVR and DBeQ treatment. Symbol **represents *p* value ≤ 0.01 in Student's *t*-test performed by SigmaPlot software.

Elongin ubiquitin ligase complex is one of the E3 ubiquitin ligases involved in UVR-induced RNAPII degradation [8, 9]. The Elongin ubiquitin ligase complex contains Cul2/Cul5, Rbx1/Rbx2 and pVHL. The latter acts as a substrate receptor for RNAPII [9–11]. The involvement of pVHL in TC-NER was suggested by the examination of TCR-NER dependent toxicity of antitumor compound Et743 [39]. Therefore, we tested if VCP/p97 is required for UVR-induced RNAPII degradation in the context of cellular pVHL background. In pVHL-deficient UOK121 cells, UVR induced a discernible loss of RNAPII at 4 to 6 h, which was prevented by VCP/p97 inhibition (Figure 7A). In pVHL-proficient UOK121-VHL cells, the level of RNAPII (Pol IIo form) was relatively higher before and 2 h after UVR treatment. Yet, a more profound UVR-induced RNAPII loss occurred. The RNAPII loss was partially rescued by VCP/p97 inhibition. Thus, both pVHL-dependent and -independent RNAPII degradation requires VCP/p97 function.

Since Elongin ubiquitin ligase complex contains Cullin (Cul2 or Cul5), and the activity of Cullin-based ubiquitin ligases is regulated by neddylation, a process that modifies Cullin with small ubiquitin-like protein Nedd8 [40]. We asked whether neddylation is required for UVR-induced RNAPII degradation in the absence of CSB. The 24-h pretreatment with neddylation inhibitor MLN2924 effectively blocked Cul4A neddylation as judged by the disappearance of Nedd8 conjugates and the accumulation of native Cul4 (Figure 7B). The neddylation inhibition increased RNAPII levels without UV irradiation, and effectively prevented UVR-induced RNAPII from degradation. Notably, this happened in the absence of CSB, further confirming the UVR-induced RNAPII degradation can take place without TC-NER attempts. To conclude, neddylation of Cullins is required for efficient UVR-induced RNAPII degradation.

Next, we investigated cell viability of pVHL-deficient UOK121 and pVHL-proficient UOK121-VHL cells in response to UVR and VCP/p97 inhibition. The UOK121-VHL cells were more resistant to UVR-induced cell killing than UOK121 cells (Figure 7C). We noticed that this UVR response was opposite of the effect caused by antitumor compound Et743 [39]. Importantly, the VCP/p97 inhibition by DBeQ exhibited obvious toxicity without UVR and significantly increased UVR toxicity in UOK121-VHL cells at doses as low as 10 J/m^2^, indicating a pVHL-dependent UVR toxicity at low UV dose under VCP/p97 inhibition. We concluded that VCP/p97 plays a role in cell survival after UVR exposure.

## DISCUSSION

**Figure 8:**
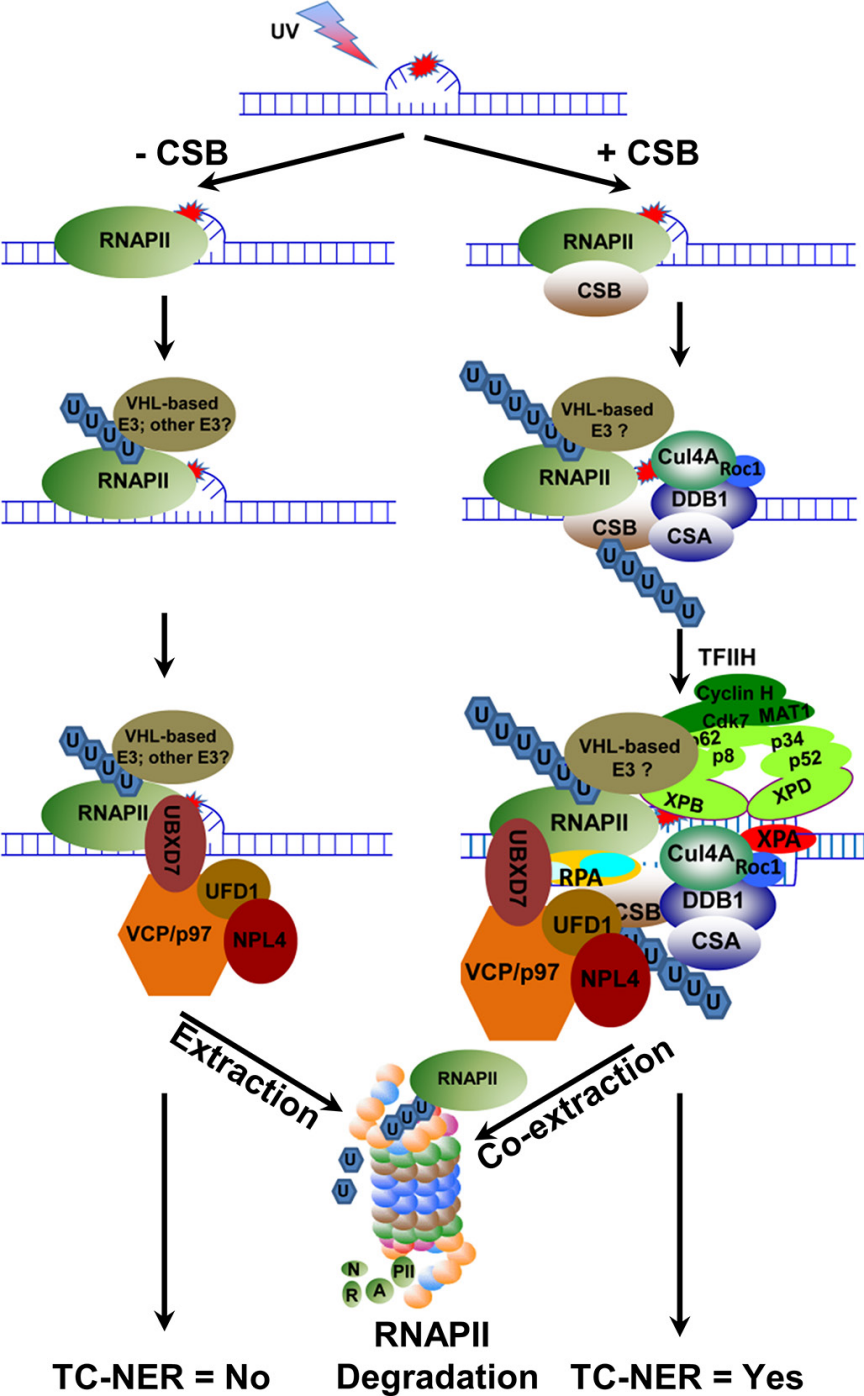
Hypothetical model depicting the role of VCP/p97 in UVR-induced RNAPII proteolysis and TC-NER When elongating RNAPII encounters a transcription-blocking DNA lesion, it arrests at the lesion sites. In the absence of CSB, the arrested RNAPII recruits E3 ubiquitin ligase(s), e.g. VHL E3 ubiquitin ligase complex, and is ubiquitinated. The ubiquitinated RNAPII attracts VCP/p97 segregase complex, which in turn extracts ubiquitinated RNAPII from chromatin and presents it to proteasome for degradation in a fast-paced manner. When CSB is present, transiently stabilized CSB-RNAPII complex recruits CRL4^CSA^, an E3 ubiquitin ligase for CSB, and VHL E3 ubiquitin ligase complex, a potentially TC-NER relevant E3 for RNAPII to ubiquitinate CSB and RNAPII, respectively. Subsequently, VCP/p97 segregase complex co-extracts ubiquitinated CSB and RNAPII from the lesion sites, which in turn facilities the sequential incorporation of arriving core NER factors into pre-incision complex. The ubiquitinated RNAPII is eventually presented to proteasome by VCP/p97 for degradation while the ubiquitinated CSB is rescued by deubiquitination.

This study provides evidence that in mammalian cells, VCP/p97 segregase complex functions in UVR-induced RNAPII degradation *via* extraction of tight chromatin-bound ubiquitinated RNAPII. The UVR-induced RNAPII degradation always requires VCP/p97 function, but can occur regardless of CSB function. Remarkably, we found that CSB enhances the RNAPII ubiquitination and the association between VCP/p97 and RNAPII, but slows down UVR-induced RNAPII degradation. These findings can be reconciled through the proposed model in which CSB plays a role in coordinating the extraction or co-extraction of ubiquitinated RNAPII and CSB itself by VCP/p97 (Figure 8). Lastly, we showed that VCP/p97 mediates UVR-induced RNAPII degradation that occurs with and without pVHL, a substrate receptor of Elongin E3 ubiquitin ligase for RNAPII ubiquitination. We inferred that the coordinated co-extraction of ubiquitinated RNAPII and CSB by VCP/p97 segregase presents an essential step of TC-NER.

RNAPII that undergoes ubiquitin-mediated degradations has two important features: the elongating and the irreversible stalling [41]. However, what makes the stalling of an elongating RNAPII irreversible is poorly understood. If degradation is an endpoint of the irreversibly stalled RNAPII, its ubiquitination in essence would be the signature of the irreversible stalling. The proteasomal degradation requires that the substrates carry at least 4 lysine 48-linked ubiquitin moieties *in vitro* [42]. The role of ubiquitin chain length in limiting the amount of time of functional action before proteolysis was first described by the study of steroid receptor coactivator-3 [43]. In our study, photolesion-stalled RNAPII is ubiquitinated more in the presence than in the absence of CSB. Yet, the UVR-induced RNAPII turnover is faster in the absence of CSB. This phenomenon is intriguing, because “the role of ubiquitin chain length” does not appear to apply to irreversibly stalled RNAPII. In earlier investigations, the effects of CS deficiency on UVR-induced RNAPII ubiquitination were intensively investigated [7, 32, 35]. Although the experiments were conducted in different cell lines and in different time frames following UVR, RNAPII ubiquitination was found somewhat compromised but not fully defective in CS cells. On the other hand, a consensus on UVR-induced RNAPII turnover requiring CSA and CSB function is still lacking [7, 38, 44]. Our comparison of UVR-induced RNAPII ubiquitination and turnover was conducted in established isogenic cell lines with inducible CSB transgene and, therefore, only introduces a single variable factor that could affect the RNAPII ubiquitination and turnover. In our study, CSB enhances RNAPII ubiquitination but slows down UVR-induced RNAPII turnover. Such a protective function of CSB on UVR-induced turnover is analogous to, as well as consistent with, the function of Rad26, a yeast homolog of CSA and CSB [45]. In yeast, Rad26 appears to antagonize Def1 in ubiquitin-mediated Rpb1 degradation. We envisage that RNAPII ubiquitination, although independent of CSB function, occurs more efficiently on stabilized RNAPII-CSB complex that is known to attract other needed proteins, *e.g*., CSA-containing E3 ubiquitin ligase, other core NER factors and non-NER factors [31]. As more proteins disembark onto the RNAPII-CSB complex, the stalling of RNAPII becomes progressively irreversible, and more ubiquitin moieties are added to RNAPII. Ultimately, the ubiquitinated RNAPII and CSB is coordinately extracted or co-extracted by VCP/p97 from chromatin for proteolysis, and for the commencement of cellular TC-NER events (Figure 8). Here, the depiction of events like ubiquitination, VCP-mediated extraction, and core NER factor recruitment is somewhat oversimplified as the participation of non-NER factors in expected to be dynamic in nature. Hence, the model only provides a tentative understanding but not the actual formation of large functioning complexes. In the case of CSB deficiency, RNAPII ubiquitination can still exceed the minimal degradable ubiquitin length and the ubiquitinated RNAPII will be quickly extracted from chromatin and undergo proteolysis. Thus, the ability of CSB to transiently stabilize RNAPII not only creates a time window but also provides protein elements for transitioning from transcription elongation to TC-NER.

The function of CSB to enhance RNAPII ubiquitination and transiently prevent RNAPII turnover appears to be in agreement with its ability to stabilize the RNAPII-CSB interaction at DNA lesions [29, 30]. The CSB's yeast homologue, Rad26, has a well-documented role in transcription elongation [46–48]. However, it remains unclear whether enhancement of RNAPII ubiquitination by CSB is related to its chromatin remodeling activity [49] and/or chromatin regulation activity [47, 50], which regulate transcription elongation. It has been shown that Rad26 is recruited to the site of DNA lesion in an elongating RNA polymerase II-dependent manner *in vivo* [48], and is required for removal of RNAPII from UVR-damaged chromatin [51]. It is possible that CSB can use its remodeling activity to displace a reversibly stalled RNAPII but not an irreversibly stalled RNAPII, which is ubiquitinated by E3(s). It is also possible that CSB ubiquitination compromises its remodeling activity, such that both ubiquitinated CSB and RNAPII have to be extracted from damaged chromatin by VCP/p97.

It has been proposed that yeast cells degrade irreversibly stalled RNAPII as a last resort for cell survival, when a transcription block, e.g., a DNA lesion, cannot be repaired or bypassed [45]. In our previous study, cellular toxicity of UVR under VCP/p97 inhibition was further exacerbated with CSB than without CSB [26]. Considering that both RNAPII and CSB are substrates of VCP/p97, it is conceivable that the clearance of ubiquitinated RNAPII from chromatin by VCP/p97 offers a better chance for cell survival both with and without TC-NER attempts. In this study, we were particularly intrigued by the findings from the investigation of VCP/p97 function in the context of pVHL-containing Elongin E3 ligase (Figure 7). The pVHL re-expression accelerates UVR-induced VCP/p97-mediated RNAPII turnover, confirming that pVHL-containing Elongin E3 ligase is at least partially responsible for RNAPII turnover. This notion is further supported by the finding that neddylation [of Cullin(s)] is required for UVR-induced RNAPII turnover. Interestingly, such a requirement of Cullin neddylation was seen in the absence of CSB, suggesting that Elongin E3 ligase can work to degrade RNAPII even without TC-NER attempts. By examining the effect of pVHL re-expression and VCP/p97 inhibition on cell viability, we revealed a potential link between TC-NER and pVHL-VCP/p97-mediated RNAPII turnover. First, pVHL re-expression confers greater UVR resistance as compared to pVHL deficiency. Importantly, such a survival advantage was not observable with antitumor compound Et743 [39]. Instead, pVHL re-expression contributes to the toxicity of Et743. It is known that Et743 toxicity requires functional TC-NER and arises from the trapping of TC-NER machinery to Et743-DNA adducts due to abortive TC-NER attempts [52]. Given that pVHL re-expression re-establishes TC-NER proficiency which contributes to Et743 toxicity [39], it is conceivable that TC-NER of photolesions contributes resistance of pVHL-proficient UOK121 cells to UVR. Second, VCP/p97 inhibition decreases cell viability regardless of pVHL but more importantly, VCP/p97 inhibition increased UV killing at low dose (10 J/m^2^) in pVHL re-expressed cells, signifying that VCP/p97 inhibition abolishes TC-NER proficiency conferred by pVHL re-expression. Thus, these findings suggest that VCP/p97-mediated extraction of RNAPII may be an integral part of TC-NER. However, the exact role of VCP/p97 in TC-NER warrants more vigorous testing in future studies.

In summary, we have described the function of VCP/p97 segregase and CSB in proteolytic clearance of RNAPII from damaged chromatin. The findings will allow further in-depth investigations of TC-NER, e.g., identification of E3 ubiquitin ligase(s) relevant to RNAPII ubiquitination in TC-NER. The findings also have clinical implications in targeting VCP/p97 for cancer therapy. Notably, VCP/p97 silencing has shown pronounced synergistic interactions with DNA-damaging agents [53]. Thus, VCP/p97 inhibitors may be more effectively used in combination with proteasome inhibitors and transcription-blocking DNA-damaging cancer therapeutics in future translational studies.

## MATERIALS AND METHODS

### Cell lines, chemicals and antibodies

HCT116 and HCT116-USP7^–/–^ cells were obtained from Vogelstein laboratory [54]. U2OS cell lines, stably transfected with tetracycline-inducible DNA constructs expressing Myc-tagged Wt or Mut EQ (E578Q)-VCP/p97, were provided by Weihl laboratory [55]. The dominant-negative EQ mutant is deficient in ATP-hydrolysis activity of VCP/p97. The CS1AN+iCSB cell line, a derivative of CSB-defective CS1AN cells, was a gift from Dr. Zhou, Yonggang [56]. CS1AN+iCSB cells express CSB under the control of the tetracycline-responsive promoter. The UOK121 (pVHL-deficient cell) and UOK121-VHL (also called UOK121wt cells) were from Dr. W. Marston Linehan laboratory. UOK121 has a hyper-methylated copy and a silent copy of VHL, and thus lacks pVHL expression; UOK121-VHL cell line has a restored pVHL expression from a stably transfected VHL cDNA [57, 58].

VCP/p97 inhibitor DBeQ, N^2^,N^4^-Dibenzylquinazoline-2,4-diamine, was purchased from Sigma-Aldrich (St. Louis, MO 63103). Proteasome inhibitor MG132 was obtained from EMB Millipore (Billerica, MA 01821).

Cul4A antibody from Bethyl Laboratories (Montgomery, TX 77356), VCP/p97 antibody from Abcam (http://www.abcam.com/), UFD1 antibody from BD Biosciences (Franklin lakes, NJ 07417), NPL4 antibody from Novus biological (Littleton, CO 80120), ubiquitin antibody FK2 from EMB Millipore, anti-Myc epitope antibody from Cell signaling (Danvers, MA 01923) and RNAPII H5 antibodies from Biolegend (San Diego, CA 92121) were obtained from their individual vendors. The anti-FLAG M2 and anti-Myc agarose affinity gels were purchased from Sigma-Aldrich. Whereas, the CSB, UBXD7 and RNAPII N20 antibodies were purchased from Santa Cruz Biotechnology (Dallas, TX 75220), RNAPII antibodies 4H8 and the antibodies against RPN2/S1 and Sug1 were purchased from Thermo Scientific (Rockford, IL 61105).

### RNA interference

All small interfering RNA (siRNA) oligonucleotides in a purified and annealed duplex form were purchased from Qiagen (Valencia, CA 91355). The targeting sequences are as follows: UBXD7 (SI00455364, catalog number), 5′-CAGCACGTGCATATTCATTTA-3′; UFD1 (SI04132583), 5′-CACTGGATGATGCAGAACTTA-3′ VCP/p97 (SI030197300), 5′-AACAGCCAUUCUCAAACAGAA-3′ and control (Ctrl) siRNA (SI03650325), 5′-AAUUCUCCGAACGUGUCACGU-3′.

### Cell culture, DNA and siRNA transfection

CS1AN cell lines, U2OS, UOK121, HeLa cells and their derivatives were grown in Dulbecco's modified Eagle medium (DMEM); HCT116 and HCT116-USP7^−/−^ cells were grown in McCoy's 5A. All cells are grown in medium supplemented with 10% fetal bovine serum (FBS), 1% penicillin and streptomycin at 37°C in a humidified atmosphere of 5% CO_2_. Antibiotics Hygromycin B and Zeocin were used for maintaining stably transfected CS1AN cell lines and U2OS cell lines as required.

DNA constructs were transfected into U2OS, HeLa or CS1AN cells using Fugene 6 transfection reagents (Promega Corporation, Madison, WI 53711), while the siRNA transfection was performed using Lipofectamine 2000 reagents (Life Technologies, Grand Island, NY 14072). All DNA and siRNA transfection experiments were conducted according to the manufacturer's protocols.

### Cellular protein fractionation

Cellular fractionation was carried out as described by Anindya et al. [7], with some modifications. The cells were lysed in ~5× cell volume of cytoplasmic lysis buffer (10 mM Tris-HCl [pH 7.9], 0.34 M sucrose, 3 mM CaCl_2_, 2 mM Mg(OAc)_2_, 1 mM DDT, 0.1 mM EDTA, 0.5% NP-40 and a protease inhibitor cocktail). Nuclei were pelleted by centrifugation at 3, 500 g for 15 min and washed with wash buffer (cytoplasmic lysis buffer without NP-40) and then lysed in ~5× cell volume of nuclear lysis buffer (20 mM HEPES [pH 7.9], 10% glycerol, 3 mM EDTA, 150 mM KOAc, 1.5 mM MgCl_2_ and protease inhibitors). The cellular nucleoplasmic fractions were separated by centrifugation at 15, 000 g for 30 min and the pellets were designated as chromatin fraction. For further processing, the pellets were re-suspended in ~1× cell volume of nuclease incubation buffer (150 mM HEPES [pH 7.9], 150 mM KOAc, 1.5 mM MgCl_2_ and protease inhibitors) and incubated with 50 U Benzonase for 30 min at room temperature. The nuclease-releasable/soluble chromatin fraction was collected by centrifugation at 20, 000 g for 30 min. The pellets were designated as insoluble chromatin fraction, and were dissolved by boiling in SDS sample buffer.

### Immunoprecipitation

The immunoprecipitation, using cell lysates or cellular protein fractions in RIPA buffer (50 mM Tris-HCl [pH 8.0], 150 mM NaCl, 1% NP-40, 2 mM EDTA, 0.5% Sodium Deoxycholate and 0.1% SDS), was done with anti- RNAPII or anti-VCP/p97 or control antibodies at 4°C overnight. The immuno-complexes were captured by protein A Plus G agarose beads. The inducible Myc-tagged Wt or EQ-VCP/p97 from cell lysates in RIPA buffer was immunoprecipitated with anti-Myc affinity gels. The proteins or ubiquitin conjugates recovered by immunoprecipitation were analyzed by Western blotting.

### Cell viability assay

The MTT [3-(4,5-Dimethylthiazol-2-yl)-2,5-Diphenyltetrazolium Bromide] assay was used to determine cell viability. UOK121 and UOK121-VHL cells were seeded in 96-well plates at proper density (7,000 cells per well) in day 1 and incubated for 24 h. In day 2, the cells were UV irradiated at variable doses from 10 to 50 J/m^2^, immediately treated with 5 μM DBeQ or vehicle, and cell cultures continued for 48 h. The cells were supplied with 100 μl fresh medium, and 10 μl of MTT stock reagent (5 mg/ml) were added directly to 96-well plates and incubated for another 4 h. Subsequently, the culture medium including MTT was carefully removed, and the cells were washed once with PBS. After removal of PBS, 200 μl of DMSO were added to each well, and the plate was gently shaken for dissolving the formazan crystals. The absorbance was read at 540 nm. The relative cell viability was calculated from the ratios of absorbance of treated to untreated cells, each from an average of 6–8 replicates.

### Quantitative analysis and statistics

Quantitative analysis was done on digitalized Western blotting images by ImageJ software and the relative protein amounts were calculated based on gray density. The Student's *t*-test was performed using SigmaPlot software.

## SUPPLEMENTARY MATERIALS FIGURES AND TABLES



## Abbreviations

RNAPII: RNA polymerase II
NER: nucleotide excision repair
GG-NER: global genomic NER
TC-NER: transcription-coupled NER
VCP/p97: Valosin-containing protein/p97
UPS: ubiquitin-proteasome systems
UVR: ultraviolet radiation
CSB: Cockayne syndrome B protein
VHL: Von Hippel-Lindau tumor suppressor
pVHL: Von Hippel-Lindau tumor suppressor protein
CSA: Cockayne syndrome A protein
TFIIH: transcription factor II H
USP7: ubiquitin specific protease 7
