# Supplementary material for: UV-induced proteolysis of RNA polymerase II is mediated by VCP/p97 segregase and timely orchestration by Cockayne syndrome B protein
Source: Oncotarget. 2016 Dec 26;8(7):11004–19. doi: 10.18632/oncotarget.14205 (PMC5355241; doi:10.18632/oncotarget.14205)
Supplement: Supplementary file 1 [file oncotarget-08-11004-s001.pdf]

UV-induced proteolysis of RNA polymerase II is mediated by VCP/p97 segregase and timely orchestration by Cockayne syndrome B protein

Supplementary Materials

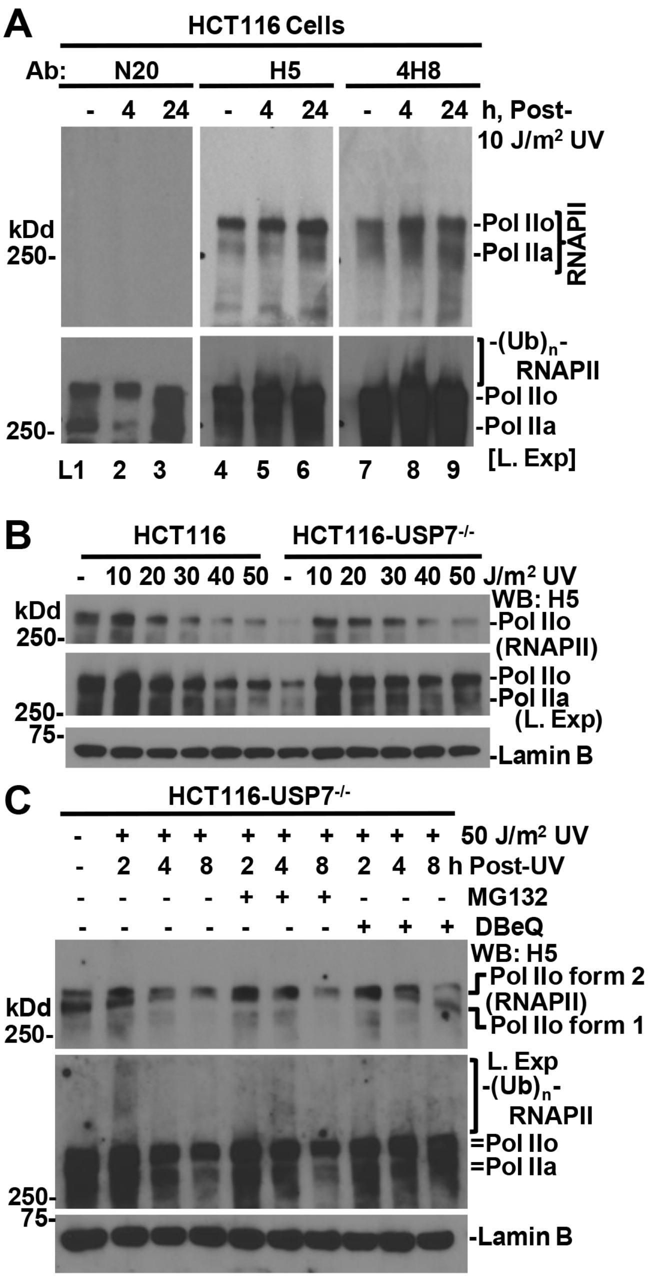

Supplementary Figure S1: (**A**) Examination of specificity and sensitivity of different RNAPII antibodies. RNAPII in lysates from HCT116 cells was analyzed by Western blotting using N20, H5, or 4H8 antibody. Longer exposure (L. Exp) was to show various forms of RNAPII. (**B**) HCT116 and HCT116-USP7$^{-/-}$ (knockout) cells were UV irradiated at different doses and maintained for 6 h. (**C**) HCT116-USP7$^{-/-}$ (knockout) cells were UV irradiated at 50 J/m^2, maintained for the indicated times with or without treatment of MG132 or VCP/p97 DBeQ at 10 µM, and the cellular proteins were analyzed by Western blotting.
